# Supplementary material for: A Novel Inducible Prophage from Burkholderia vietnamiensis G4 Is Widely Distributed across the Species and Has Lytic Activity against Pathogenic Burkholderia
Source: Viruses. 2020 May 31;12(6):601. doi: 10.3390/v12060601 (PMC7354579; doi:10.3390/v12060601)
Supplement: Supplementary file 1 [file viruses-12-00601-s001.zip › viruses-806493-suppl.pdf]

viruses

MDPI

# Supplementary materials

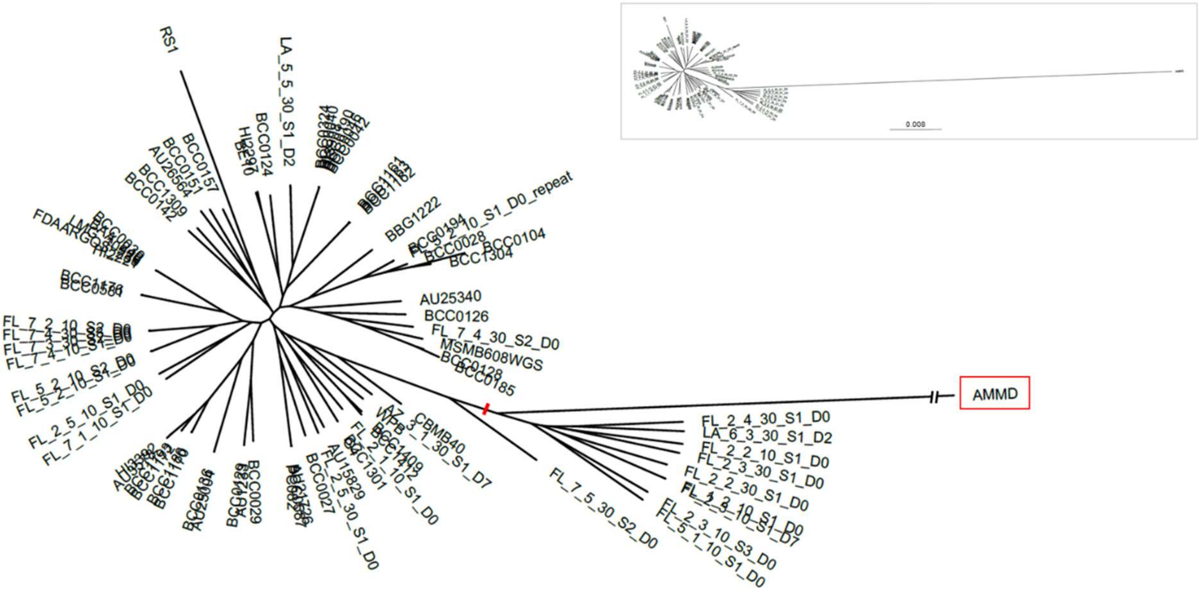


**Figure S1. Core-gene phylogeny of 81 *B. vietnamiensis* strains rooted with the outgroup *B. ambifaria* AMMD.** A maximum-likelihood phylogenetic tree based on the alignment of 1510 shared 'core' genes. The outgroup *B. ambifaria* AMMD is shown by the red box and the position of the root for the *B. vietnamiensis* core-gene phylogeny (Figure 6) is shown by the short red line. The outgroup was shortened in the main figure for visualisation purposes; a framed miniature of the true appearance of the tree is shown at the top right, the scale bar indicates the evolutionary distance as number of base substitutions per site.

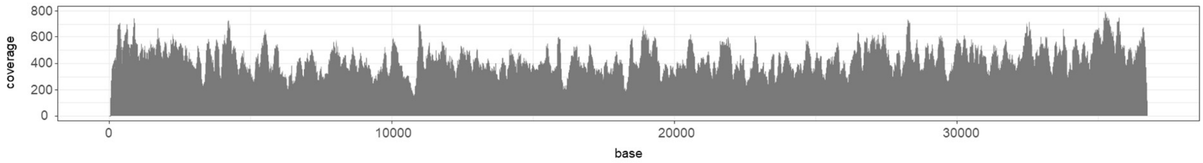


**Figure S2. Depth of sequencing coverage plot for vB_BvM-G4P1.** The x-axis displays the position in the phage genome assembly and the y-axis is the coverage.

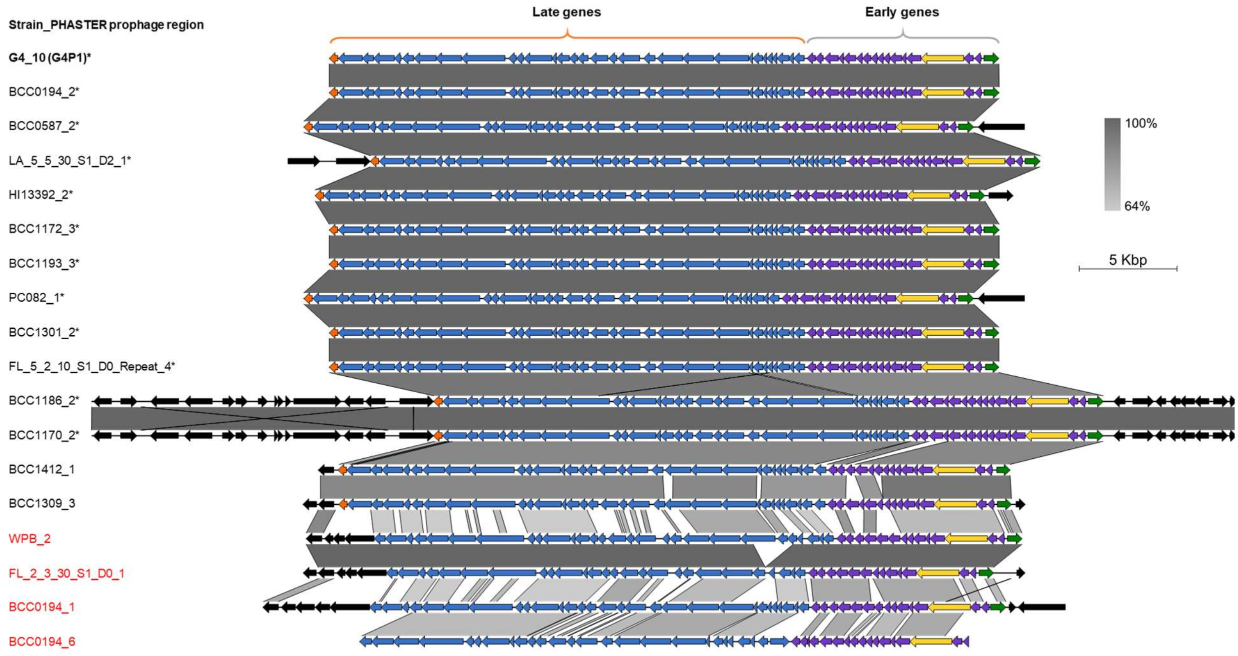


**Figure S3. Similarities in the genome organisation of the G4P1, G4P1-like and G4P1-variant prophage regions identified by PHASTER.** EasyFig was used to visualize the comparison of G4 prophage region 10 (G4P1 without the beta-galactosidase gene) to the 13 G4P1-like and 4 G4P1-variant prophage regions identified by PHASTER. The comparison revealed a shared backbone with two sub-regions, broadly comprising early (grey bracket; purple arrows) and late (orange bracket; blue arrows) genes. G4P1 start (tail fiber assembly protein; orange arrow) and end (putative phage cI repressor; green arrow) genes are shown but were not always present in the G4P1-variant prophages. Each prophage encoded a transposase gene of 2.2 kb in length within the early region (yellow arrow). Genes outside the shared region are shown by black arrows. Strains have been organized from highest (top) to lowest (bottom) %ANI similarity to G4 prophage region 10 and prophage region identifiers are shown to the left of the alignment, with G4P1-variants highlighted in red. Genome length is indicated by the scale line (bp) to the right of the alignment. An asterisk (*) next to the strain name indicates the presence of a beta-galactosidase gene upstream of the tail fiber assembly protein in the lysogen genome. The grey vertical blocks between prophage sequences display %blastn shared similarity and the degree of sequence similarity is indicated by the gradient scale.

**Table S1.** *B. vietnamiensis* strains, genomes and prophage regions identified by PHASTER (separate Excel spreadsheet)

**Table S2.** Summary of vB_BvM-G4P1 sequencing and genome assembly results

| FastQC output (reads) | | QUAST output (assembly) | | Average sequencing coverage (Reads mapped onto phage assembly) |
|---|---|---|---|---|
| Read N° (trimmed) | Average read length (trimmed) (bp) | Assembled phage length (bp) | GC (%) | |
| 73473 | 122 (20-151) | 36752 | 62.51 | 442 |

viruses

MDPI

1

2 **Table S3.** Gene annotations for the chromosomal location of the vB_BvM-G4P1 prophage region in *B. vietnamiensis* G4

| ***B. vietnamiensis* G4 chromosome 1 gene designation** | ***B. vietnamiensis* G4 chr 1 *Burkholderia* genome database annotation** | **vB_BvM-G4P3 Prokka (*Caudovirales* database) annotation** | **PHASTER annotation** | **Interpro predicted protein family membership (predicted domains)** | **Final annotation** |
|---|---|---|---|---|---|
| R0040 | tRNA-Arg | - | - | - | - |
| Bcep1808_1284 | Beta-galactosidase | Beta-galactosidase | - | - | Beta-galactosidase |
| Bcep1808_1285 | Hypothetical protein | Tail fiber assembly protein | Tail fiber assembly protein | Phage tail assembly chaperone protein IPR031893 | Tail fiber assembly protein |
| Bcep1808_1286 | Hypothetical protein | Phage tail fiber protein | Phage tail fiber protein | None predicted | Tail fiber protein |
| Bcep1808_1287 | Hypothetical protein | Tail protein | Tail protein | Bacteriophage Mu-like, Gp48 IPR018755 | Tail protein |
| Bcep1808_1288 | Baseplate J family protein | Baseplate J family protein gp47 | Baseplate J protein | Baseplate J like IPR006949 | Baseplate J protein |
| Bcep1808_1289 | Phage GP46 family protein | Mu bacteriophage protein gp46 | Hypothetical protein | Bacteriophage Mu, Gp46 IPR010877 | Hypothetical protein |
| Bcep1808_1290 | Phage baseplate assembly protein V | Phage-related baseplate assembly protein gp45 | Baseplate assembly protein | Phage baseplate assembly protein V/Gp45 IPR013046 and Phage-associated protein Gp45 IPR014462 (phage spike domain IPR040629) | Baseplate assembly protein |
| Bcep1808_1291 | Bacteriophage Mu P family protein | Mu-like prophage tail protein gpP | Tail protein | Baseplate protein GpP IPR026276 | Tail protein |
| Bcep1808_1292 | DNA circulation family protein | DNA circulation protein | DNA circularisation protein | None predicted (DNA circulation domain IPR009826) | DNA circulation protein |

| | | | | | |
|---|---|---|---|---|---|
| Bcep1808_1293 | TP901 family phage tail tape measure protein | Bacteriophage tail length determination protein | Tail length tape measure protein | None predicted (Phage tail tape measure protein domain IPR010090) | Tail tape measure protein |
| Bcep1808_1294 | Hypothetical protein | Hypothetical protein | Hypothetical protein | None predicted | Hypothetical protein |
| Bcep1808_1295 | Hypothetical protein | Tail tube protein | Tail tube protein | None predicted | Tail tube protein |
| Bcep1808_1296 | Bacteriophage Mu tail sheath family protein | Tail sheath protein gpL | Tail sheath protein | Tail sheath protein IPR007067 | Tail sheath protein |
| Bcep1808_1297 | Hypothetical protein | Hypothetical protein | Hypothetical protein | Protein of unknown function DUF2635 IPR024400 | Hypothetical protein |
| Bcep1808_1298 | Hypothetical protein | Mu-like prophage protein gp37 | Hypothetical protein | Bacteriophage Mu, Gp37 IPR014972 | Hypothetical protein |
| Bcep1808_1299 | Hypothetical protein | Mu-like prophage protein gp36 | Hypothetical protein | Bacteriophage Mu, Gp36 IPR009752 | Hypothetical protein |
| Bcep1808_1300 | Hypothetical protein | Hypothetical protein | Mu phage uncharacterised protein | None predicted | Hypothetical protein |
| Bcep1808_1301 | Mu-like prophage major head subunit gpT | Major head subunit | Head protein | None predicted (Bacteriophage Mu GpT domain IPR018774) | Head protein |
| Bcep1808_1302 | Hypothetical protein | Hypothetical protein | Bacteriophage protein | None predicted | Hypothetical protein |
| Bcep1808_1303 | Hypothetical protein | Protease (I) and scaffold (Z) proteins | Mu-like prophage I protein | Protease, Mu phage/prophage I type IPR012106 | Protease |
| Bcep1808_1304 | Phage virion morphogenesis protein | gp31 | G protein 2 | Phage virion morphogensis protein IPR006522 | Virion morphogensis protein |
| Bcep1808_1305 | SPP1 family phage head morphogenesis protein | gp30 | Putative virion morphogenesis protein | None predicted (Phage head morphogenesis domain IPR006528) | Virion morphogensis protein |
| Bcep1808_1306 | Hypothetical protein | Portal protein | Portal protein | Protein of unknown function DUF935 IPR009279 | Portal protein |

| | | | | | |
|---|---|---|---|---|---|
| Bcep1808_1307 | Hypothetical protein | Large terminase | Hypothetical protein | None predicted | Large terminase (due to similarities in organisation to vB_MhM-3927AP2) |
| Bcep1808_1308 | Hypothetical protein | Hypothetical protein | Hypothetical protein | None predicted | Hypothetical protein |
| Bcep1808_1309 | DNA-binding protein | DNA-binding protein | Hypothetical protein | Bacteriophage D3112, Orf24 IPR014926 | DNA-binding protein |
| Bcep1808_1310 | Hypothetical protein | Hypothetical protein | Hypothetical protein | None predicted | Hypothetical protein |
| Bcep1808_1311 | Hypothetical protein | Hypothetical protein | Hypothetical protein | None predicted | Hypothetical protein |
| Bcep1808_1312 | TraR/DksA family transcriptional regulator | DksA-like zinc finger domain containing protein | DksA-like zinc finger domain containing protein | None predicted (Zinc finger, DksA/TraR C4-Type domain IPR000962) | DksA-like zinc finger domain containing protein; putative transcriptional regulator |
| Intergenic spacer | Intergenic spacer | Putative Rz lysis protein | Putative Rz lysis protein | None predicted | Hypothetical protein |
| Bcep1808_1313 | Hypothetical protein | Hypothetical | Hypothetical protein | None predicted | Hypothetical protein |
| Bcep1808_1314 | Glycoside hydrolase family protein | Putative endolysin protein | Putative phage lysozyme | Glycoside hydrolase, family 24 IPR002196 | Lysis protein |
| Bcep1808_1315 | Hypothetical protein | Putative integral membrane protein | Mor transcription activator family protein | None predicted (Mor transcription activator domain IPR014875) | Mor transcription activator |
| Bcep1808_1316 | Hypothetical protein | gp02 | Hypothetical protein | Bacteriophage Mu, Gp16 IPR009363 | Hypothetical protein |
| Bcep1808_1317 | Putative phage associated protein | Hypothetical protein | Hypothetical protein | Bacteriophage B3, Orf5 IPR016868 (DUF2786 IPR024498) | Hypothetical protein |
| Bcep1808_1318 | Hypothetical protein | Hypothetical protein | Hypothetical protein | None predicted | Hypothetical protein |
| Bcep1808_1319 | Hypothetical protein | Product=gp36 | gp05 | Bacteriophage B3, Orf6 IPR021505 | Hypothetical protein |
| Bcep1808_1320 | Hypothetical protein | Hypothetical protein | Hypothetical protein | None predicted | Hypothetical protein |
| Bcep1808_1321 | Hypothetical protein | Hypothetical protein | Ren protein | None predicted | Hypothetical protein |
| Bcep1808_1322 | Hypothetical protein | Hypothetical protein | Hypothetical protein | None predicted | Hypothetical protein |
| Bcep1808_1323 | Hypothetical protein | Hypothetical protein | Hypothetical protein | None predicted | Hypothetical protein |

| | | | | | |
|---|---|---|---|---|---|
| Bcep1808_1324 | Regulatory protein LacI | LacI-like regulatory protein | Hypothetical protein | None predicted (LacI-type HTH domain IPR000843) | LacI-like regulatory protein |
| Bcep1808_1325 | Hypothetical protein | Hypothetical protein | Hypothetical protein | None predicted | None predicted |
| Bcep1808_1326 | Hypothetical protein | Hypothetical protein | Hypothetical protein | None predicted | None predicted |
| Bcep1808_1327 | Hypothetical protein | DNA transposition protein | Putative DNA transposition protein | None predicted (AAA+ ATPase domain IPR003593) | DNA transposition protein |
| Bcep1808_1328 | MuA transposase/repressor protein CI, DNA binding | A transposase | A transposase | Transposase InsF-like IPR038965 (integrase catalytic core domain IPR001584) | A transposase |
| Bcep1808_1329 | Hypothetical protein | gp43 | gp13 | None predicted | Hypothetical protein |
| Bcep1808_1330 | Putative transcriptional regulator, Nlp | Mu-like prophage FluMu DNA-binding protein Ner | Mu-like prophage FluMu DNA-binding protein Ner | None predicted (Cro/C1-type helix-turn-helix domain IPR001387) | Mu-like prophage FluMu DNA-binding protein Ner |
| Bcep1808_1331 | Putative phage repressor | Putative cI repressor | Protector from prophage-induced early lysis | None predicted (Cro/C1-type helix-turn-helix domain IPR001387 and LexA-like domain IPR039418) | Putative cI repressor |

**Table S4.** Comparison of G4P1, the G4P1-like and G4P1-variant prophage regions

| Strain_prophage region* | ANIb (G4_10 query) | blastn results (G4_10 query) | | | | | | | | | | tblastx results (G4_10 query) | | | | | | | | | |
|---|---|---|---|---|---|---|---|---|---|---|---|---|---|---|---|---|---|---|---|---|---|
| | | % Identity | Alignment length | Mismatches | Gap openings | Query start | Query start | Subject start | Subject send | E-value | Score | % Identity | Alignment length | Mismatches | Gap openings | Query start | Query start | Subject start | Subject send | E-value | Score |
| **G4_10** | 1 | 100 | 34252 | 0 | 0 | 1 | 34252 | 1 | 34252 | 0 | 63252 | 100 | 822 | 0 | 0 | 32681 | 30216 | 32681 | 30216 | 0 | 1873 |
| **BCC0194_2** | 0.992054 | 99.052 | 34284 | 282 | 30 | 1 | 34252 | 1 | 34273 | 0 | 61470 | 100 | 822 | 0 | 0 | 32681 | 30216 | 32702 | 30237 | 0 | 1873 |
| **BCC0587_2** | 0.991932 | 99.064 | 34284 | 278 | 30 | 1 | 34252 | 1 | 34273 | 0 | 61492 | 100 | 822 | 0 | 0 | 32681 | 30216 | 32702 | 30237 | 0 | 1873 |
| **LA_5_5_30_S1_D2_1** | 0.991931 | 99.064 | 34284 | 278 | 30 | 1 | 34252 | 34273 | 1 | 0 | 61492 | 100 | 822 | 0 | 0 | 32681 | 30216 | 1572 | 4037 | 0 | 1873 |
| **HI3392_2** | 0.991764 | 99.043 | 34284 | 285 | 30 | 1 | 34252 | 35715 | 1443 | 0 | 61453 | 100 | 822 | 0 | 0 | 32681 | 30216 | 3014 | 5479 | 0 | 1873 |
| **BCC1172_3** | 0.989339 | 99.035 | 34285 | 286 | 30 | 1 | 34252 | 34273 | 1 | 0 | 61437 | 99.878 | 822 | 1 | 0 | 32681 | 30216 | 1572 | 4037 | 0 | 1870 |
| **BCC1193_3** | 0.989339 | 99.035 | 34285 | 286 | 30 | 1 | 34252 | 34273 | 1 | 0 | 61437 | 99.878 | 822 | 1 | 0 | 32681 | 30216 | 1572 | 4037 | 0 | 1870 |
| **PC082_1** | 0.988477 | 99.064 | 34284 | 278 | 30 | 1 | 34252 | 36875 | 2603 | 0 | 61492 | 100 | 822 | 0 | 0 | 32681 | 30216 | 4174 | 6639 | 0 | 1873 |
| **BCC1301_2** | 0.977624 | 97.93 | 34305 | 606 | 77 | 1 | 34246 | 1 | 34260 | 0 | 59321 | 99.887 | 883 | 1 | 0 | 2418 | 5066 | 2439 | 5087 | 0 | 1865 |
| **FL_5_2_10_S1_D0_repeat_4** | 0.964073 | 96.575 | 34311 | 1051 | 94 | 1 | 34246 | 1 | 34252 | 0 | 56737 | 99.887 | 883 | 1 | 0 | 2418 | 5066 | 2439 | 5087 | 0 | 1865 |
| **BCC1186_2** | 0.909481 | 91.534 | 21487 | 1736 | 50 | 6 | 21448 | 17514 | 38961 | 0 | 29525 | 94.526 | 822 | 45 | 0 | 32681 | 30216 | 50209 | 47744 | 0 | 1761 |
| **BCC1170_2** | 0.907301 | 91.534 | 21487 | 1736 | 50 | 6 | 21448 | 41101 | 19654 | 0 | 29525 | 94.526 | 822 | 45 | 0 | 32681 | 30216 | 8406 | 10871 | 0 | 1761 |
| **BCC1412_1** | 0.893394 | 91.563 | 20694 | 1677 | 47 | 785 | 21450 | 1785 | 22437 | 0 | 28478 | 89.39 | 820 | 87 | 0 | 32681 | 30222 | 33833 | 31374 | 0 | 1673 |
| **BCC1309_3** | 0.871176 | 87.617 | 15747 | 1827 | 80 | 973 | 16645 | 34161 | 18464 | 0 | 18162 | 93.552 | 822 | 53 | 0 | 32681 | 30216 | 2262 | 4727 | 0 | 1754 |
| **WPB_2** | 0.707995 | 79.811 | 1273 | 233 | 20 | 28040 | 29302 | 30438 | 31696 | 0 | 905 | 80.036 | 561 | 112 | 0 | 21462 | 19780 | 23993 | 22311 | 0 | 1086 |
| **FL_2_3_30_S1_D0_1** | 0.718799 | 79.811 | 1273 | 233 | 20 | 28040 | 29302 | 29137 | 30395 | 0 | 905 | 66.414 | 527 | 177 | 0 | 32486 | 30906 | 33554 | 31974 | 0 | 818 |
| **BCC0194_1** | 0.748357 | 75.966 | 5022 | 1049 | 135 | 7345 | 12275 | 29980 | 25026 | 0 | 2442 | 88.007 | 592 | 71 | 0 | 21516 | 19741 | 15907 | 17682 | 0 | 1248 |
| **BCC0194_6** | 0.746752 | 75.662 | 9097 | 1971 | 207 | 3737 | 12699 | 31155 | 22168 | 0 | 4309 | 86.18 | 521 | 72 | 0 | 21312 | 19750 | 13595 | 15157 | 0 | 1120 |

Footnotes: ANIb, blastn and tblastx performed with region G4_10 as the query sequence; *prophage region as identified by PHASTER

**Table S5:** Sequences with highest similarity to the vB_BvM-G4P1 transposase identified within Genbank by blastp

| Description | Max score | Total score | Query coverage | E-value | Percentage identity | Accession |
|---|---|---|---|---|---|---|
| Transposase [*Rhizobium* phage RR1-B] | 336 | 336 | 82% | 5.00E-104 | 34.56 | YP_008129821.1 |
| Phage transposase [*Haemophilus* phage SuMu] | 307 | 307 | 84% | 5.00E-94 | 33.12 | YP_007002911.1 |
| Transposase [*Mannheimia* phage vB_MhM_3927AP2] | 275 | 275 | 88% | 4.00E-82 | 32.34 | YP_009196052.1 |
| Transposase/ integrase [*Klebsiella* phage ST405-OXA48phi1.1] | 236 | 236 | 81% | 9.00E-68 | 31.38 | QBA84945.1 |
| Transposase [*Curvibacter* phage TJ1] | 153 | 153 | 69% | 5.00E-39 | 29.23 | QDB70122.1 |
| Putative transposase [*Burkholderia* phage KS10] | 153 | 153 | 76% | 7.00E-39 | 27.75 | YP_002221432.1 |
| Integrase [*Pasteurella* phage AFS-2018a] | 94.7 | 94.7 | 51% | 2.00E-19 | 26.11 | AWY03226.1 |
| Transposase A [*Bacillus* phage BalMu-1] | 91.3 | 91.3 | 50% | 2.00E-18 | 25.57 | YP_009276812.1 |
| Transposase [*Escherichia* phage Mu] | 85.9 | 85.9 | 74% | 1.00E-16 | 23.79 | NP_050607.1 |
| Transposase [*Shigella* phage SfMu] | 73.2 | 73.2 | 76% | 8.00E-13 | 22.66 | YP_009152189.1 |
